# Selection for social genetic effects in purebred pigs improves behaviour and handling of their crossbred progeny

**DOI:** 10.1186/s12711-023-00828-9

**Published:** 2023-07-25

**Authors:** Bjarne Nielsen, Lizette Vestergaard Horndrup, Simon P. Turner, Ole Fredslund Christensen, Hanne Marie Nielsen, Birgitte Ask

**Affiliations:** 1 Center for Quantitative Genetics and Genomics, Aarhus University, C. F. Møllers Allé 3, 8000 Aarhus C, Denmark; 2 Breeding and Genetics, Pig, Danish Agriculture and Food Council F.M.B.A., Axelborg, Axeltorv 3, 1609 Copenhagen V, Denmark; 3 Animal and Veterinary Sciences, SRUC, Roslin Institute Building, Easter Bush, Midlothian, EH25 9RG UK

## Abstract

**Background:**

In commercial pig production, reduction of harmful social behavioural traits, such as ear manipulation and tail biting, is of major interest. Moreover, farmers prefer animals that are easy to handle. The aim of this experiment was to determine whether selection on social breeding values (SBV) for growth rate in purebred pigs affects behaviour in a weighing crate, lesions from ear manipulation, and tail biting of their crossbred progeny. Data were collected on crossbred F1 pigs allocated to 274 pens, which were progeny of purebred Landrace sows and Yorkshire boars from a DanBred nucleus herd.

**Results:**

Behaviour in the weighing crate scored on a three-level scale showed that groups of pigs with high SBV for growth rate were significantly calmer than groups of pigs with low SBV (P < 0.027). When the mean SBV in the group increased by 1 unit, the proportion of pigs that obtained a calmer score level was increased by 14%. A significant (p = 0.04), favourable effect of SBV was found on both the number of pigs with ear lesions in the group and the mean number of ear lesions per pig. For a 1 unit increase in mean SBV, the mean number of lesions per pig decreased by 0.06 from a mean of 0.98. Individual severity of ear lesions conditional upon the number of ear lesions was also significantly affected (p = 0.05) by the mean SBV in the group. In groups for which the mean SBV increased by 1 unit, the proportion of pigs that were observed with a lower severity score was increased by 20% on a three-level scale. Most pigs received no tail biting injuries and no effect of SBV was observed on the tail injury score.

**Conclusions:**

After 7 weeks in the finisher unit, crossbred progeny with high SBV were calmer in the weighing crate and had fewer ear lesions. These results indicate that selection of purebred parents for SBV for growth rate will increase welfare in their crossbred progeny by decreasing the number of ear lesions and making them easier to handle.

## Background

In commercial pig production, reducing the expression of harmful social behavioural traits, such as ear manipulation and tail biting and increasing the positive response to human handling, are of major interest. Ear manipulation and tail biting represent the main forms of harmful social behaviour in pigs [1], as they cause injuries, suppress immunocompetence, decrease growth rate and feed efficiency, and increase the risk of mortality [2]. As such, they significantly compromise animal welfare and economic and environmental sustainability. However, reducing the impacts of these behaviours on animal welfare and productivity is constrained by the costs of implementing management changes on-farm. One solution would be to reduce harmful social interactions by genetic selection. Social behavioural traits are, however, difficult to improve through direct genetic selection as they are costly to phenotype on a large number of selection candidates [3, 4].

Social breeding values (SBV) for growth rate have been advocated as a way to improve social behaviour [5]. The SBV is the heritable effect of an individual’s genotype on the phenotypic trait value of its group mates [6, 7]. In pigs, selection experiments performed in experimental herds with purebreds have shown that the social behaviour of the progeny of pigs with positive SBV for growth performance is improved [8, 9]. Specifically, the study by Camerlink et al. [8] reported a reduction in ear and tail injuries in groups of pigs that have a positive SBV for growth, and their results also show opportunities to reduce harmful biting behaviours in pigs. However, these results were not confirmed for other forms of biting behaviour by Canario et al. [10], who concluded that, within three weeks after mixing, selection for growth rate during the fattening period based on both direct breeding values (DBV) estimated with a classical animal model and SBV did not lead to radical changes in aggressiveness compared to selecting only on DBV for growth. Thus, although there is some evidence that key traits related to harmful social behaviour may be improved by selection on SBV [8, 9], it is not conclusive and further research is needed. In addition, improving behavioural traits in pigs may affect their response to handling in a positive direction [11] and it is important that changes in social behaviour do not compromise handling ease.

Commercial sows are mainly produced by a two-way cross of purebred lines, whereby crossbred sows benefit from heterosis [12, 13]. Phenotypes of purebred and crossbred pigs can also be affected by the environmental conditions, which differ between purebred and crossbred production systems. Wei and van der Werf [14] reported how the use of crossbred information in genetic evaluation of purebred lines maximizes genetic response in crossbreds, and many other studies have investigated the combination of purebred and crossbred performance [15, 16]. Recently, Ask et al. [17] showed in a large-scale selection experiment that an increase in the SBV for growth in purebreds increases the growth performance of their crossbred progeny. However, using SBV for growth to select for improved social behaviour requires confirmation that selection for SBV in purebreds does indeed improve social behaviour of crossbred progeny managed under commercial conditions. If the SBV for growth rate is successfully increased by selection in the purebred lines, then the mean SBV of the future groups of pigs will increase, and thus it is necessary to investigate if the behavioural traits differ between groups of pigs with high versus low SBV for growth rate.

The objective of this study was to identify if and how selection on SBV for growth rate in two purebred dam line populations had an effect on ear lesions, tail biting lesions, and response to handling in crossbred progeny managed under commercial conditions. This effect was investigated in crossbred progeny and behavioural traits were recorded twice, once after one day and once after seven weeks in the finisher unit. We hypothesised that selection on SBV for growth rate in purebreds could decrease the number and the severity of lesions from ear manipulation and tail biting of crossbred pigs, and also improve their behaviour in a weighing crate, both one day and seven weeks after entering the finisher unit. If this is the case, then selection on SBV in purebred pigs is expected to increase welfare and ease the handling of commercial crossbred pigs managed under commercial conditions.

## Methods

To investigate the effect of selection on estimates of SBV for growth rate on injuries due to social interactions and on handling ease, a large single-generation selection experiment was conducted with pens that had pigs of either low or high predicted SBV. The experiment was designed as a block design with four pens of combinations based on predicted SBV (high and low predicted SBV) and predicted DBV (high and low predicted DBV) within each block. The experiment lasted 72 weeks and each week was treated as a block. The experimental conditions are fully described in [17].

### Production of crossbred pigs and housing conditions

A selection experiment was conducted at a nucleus herd by crossing purebred DanBred Landrace (L) sows with purebred DanBred Yorkshire (Y) AI boars to produce F1 crossbred (YL) piglets during the period from October 2016 until August 2018. In total, 199 Y-boars and 911 L-sows were selected with either high or low SBV for average daily gain (ADG) and produced 1171 YL litters. Weekly, SBV and DBV for ADG were predicted within each of the two purebred populations, L and Y, using data from the routine DanBred breeding programme for the genetic evaluation of ADG recorded during performance testing of young pigs of both sexes during the growth period from 30 to 100 kg body weight. Predicted SBV and DBV for ADG were obtained from Model 2 in Ask et al. [17], which also provides a detailed description of the experimental design. Litters with high SBV were produced by mating boars and sows with high SBV and, conversely, litters with low SBV were produced by mating boars and sows with low SBV. Resulting litter mates had identical predicted breeding values that were equal to the mean of their parents’ predicted breeding values. On average, 5.3 boars (ranging from 1 to 9) and 13.1 sows (ranging from 7 to 20) were represented within each week. The mean within week selection differential of SBV between the high and low groups was 2.1 for the Y-boars and 1.1 for the L-sows, and the within-group standard deviations were 0.36 for the Y-boars and 0.25 for the L-sows. For the predicted SBV, the average value was 1.36 for crossbred progeny pigs with high SBV and -0.24 for those with low SBV, which resulted in an average selection differential of 1.60 g/day/pig for SBV.

At farrowing, the L sows were housed in individual farrowing crates, and within 24 h after farrowing, all crossbred (YL) piglets were ear-tagged with an individual identification number and a coloured ear tag to indicate high or low SBV. All staff and technicians were blinded to the meaning of the colour codes. All male piglets were castrated and used in the experiment. Female pigs were bred to be production sows and therefore, were not available for the experiment. Throughout the nursing period, piglets with high SBV were always kept separately from piglets with low SBV, even when cross-fostering or nurse sows were used. Cross-fostering was managed by the staff at the herd and was not influenced by the level of SBV.

Pigs were weaned once per week in separate groups of high and low SBV and transported to a weaner unit on a single commercial finisher farm. The pigs with high or low SBV were transported to the weaner unit in the same truck but in separate groups. In the weaning unit, each weekly weaned group of pigs was housed in a separate herd-section. Within a weekly weaned group of pigs, the weaning age could vary by several weeks due to the cross-fostering described above and because body weight was standardized within a week. After arriving at the weaner unit, all weaners with the same colour of ear-tag were placed in a single weaner pen. Thus, there were two experimental weaner pens per week i.e., one with high and one with low SBV. To equalise group size between the experimental weaner pens and to reduce variation in weight, the smallest pigs were removed from the experimental weaner pens in the following days. The two experimental weaner pens included pigs that showed maximum variation in SBV (high vs low) between pens and minimum variation in weight within pens. The average group size of the experimental weaner pens was 40.3 pigs per pen (ranging from 23 to 45 pigs).

At an average weight of 34.0 kg, pigs were transferred to the finisher unit on the same farm. Based on high and low DBV, the weaner groups were divided into two sub-groups and housed in separate finisher pens to have 18 pigs per pen. Thus, four pens were created: high SBV and high DBV; high SBV and low DBV; low SBV and high DBV; and low SBV and low DBV. Unfortunately, due to the limited number of pigs available during some weeks affected by weekly variation in the number of litters and the litter sizes, the allocation into sub-groups was unbalanced. The experimental design was incomplete during 15 of the 72 weeks, with only three experimental groups per week. However, there was always at least one pen for each of the two SBV scenarios for each week. Group size in the finisher unit averaged 17.4 pigs per pen (ranging from 12 to 19 pigs) and space allowance per pig averaged 0.82 m^2^/pig (ranging from 0.63 to 1.00 m^2^).

If a pig was physically removed from a pen, the removal date and reason (death, severe lesions, lameness, long term illness, or tail bites) were recorded; thus, in the high and low SBV groups, 2.4 and 1.8%, respectively, of the total number of pigs (4.2%) had no final body weight record due to their removal. Pigs were not allowed to re-enter their pen after having been removed, or to be transferred, neither among the experimental pens, nor from non-experimental pens to experimental pens. The experiment included 274 finisher pens.

Pigs were fed on a standard finisher diet, that fulfilled the minimum Danish requirements [18], with wet feed three times per day throughout the finisher period. Straw was provided in hanging racks on a daily basis and throughout the day, and pens had 50% solid and 50% slatted floors. Ventilation was semi-natural with automatic curtain ventilation on each side of the barn and exhaust fans in the ceiling.

### Recording of traits

Individual body weight and behavioural traits of the pigs were recorded by a technician at 24 h and again at seven weeks after transfer to the finisher unit (on average 48.2 days, ranging from 35 to 53 days).

#### Ear lesions

The total number of ear lesions per pig was recorded as the sum of the lesions on each ear. From these records, a five-category score ranging from 1 to 5 for each pig was established, corresponding to 0, 1, 2, 3, and > 3 lesions per pig. Severity of the ear lesions was also scored, using a modified version of the system by Lahrmann et al. [19] (see Table 1). If a pig had multiple ear lesions with different severities, the technician scored the most severe lesion.

**Table 1:** Severity score for ear lesions and tail biting injuries

Score	Definition
1	No injury recorded
2	Red or 1 to 2 small bite marks
3	More than two bite marks
4	Wound in which individual bite marks have become amalgamated into a damaged area of tissue

For ear lesions, if the pig had > 1 ear lesion, then only the most severe ear lesion on the pig was scored

#### Tail biting injuries

First, pigs were recorded for the presence or absence of tail biting injuries, and when tail biting injuries were present, their severity was scored using the same scores as for ear lesions (see Table 1).

#### Behaviour in the weighing crate

Animal behaviour due to human handling was scored during weight recording in the weighing crate, using a modified version of the scale by D’Eath et al. [11] that includes four categories, as shown in Table 2. Scores 3 and 4 were combined in the analyses due to the very limited number of pigs receiving a score of 4.

**Table 2:** Scoring key for behavioural responses to handling in a weighing crate

Score	Definition
1	Pig stands still during weighing and appears calm
2	Pig moves around during weighing
3	Pig moves around a lot during weighing, jumping and crashing into crate fittings
4	Pig appears ‘frozen with fear’ and stands still during weighing

### Statistical analyses

The behavioural traits were recorded individually on each pig but the experiment was designed with groups (pens) of either low or high SBV in combination with groups (pens) of DBV and, therefore, the experimental unit was regarded as a group of pigs housed in a pen. Thus, the variance between groups (pens) included the variance of the random effect used for testing the effect of SBV and DBV. Analyses at the individual pig level also considered the variation between pigs within groups (pens), but in the test of the effects of SBV and DBV, the variance between groups still needed to be estimated and considered. The advantage of conducting the analyses at the individual pig level is that the categorical recordings can be used directly, which, if more than two levels are recorded, allows the use of an ordered scale. Ear lesions and tail biting injuries were analysed at both the group and individual levels, whereas the behaviour in the weighing cate was only analysed at the individual level because the trait was recorded on an ordinal score scale and the calculation of a mean across pigs with different score values is not interpretable.

#### Group level analyses

At the group level, only one observation per pen at a given time was considered for each trait. This means that all observations within a group were aggregated into a single value. For ear lesions, two traits were considered: (1) the mean number of pigs in a group with ear lesions and (2) the mean number of ear lesions per pig within the group. For tail biting, only the mean number of pigs in the group with tail biting injuries was analysed.

Let ${\mathrm{Y}}_{j}$ be the aggregated value for group $j$ and the model given by:

1 $${\mathrm{Y}}_{j}=\,\mu +{\alpha }_{j,t}+{\delta }_{j}+{\beta }_{n}{n}_{j}+{\beta }_{age}{\tau }_{j}+{\beta }_{w}{w}_{w,j}+{\beta }_{sd}{w}_{sd,j}+{\beta }_{d}{a}_{d,j}+{\beta }_{s}{a}_{s,j}+{\beta }_{ssd}{\delta }_{s,sd,j}+{e}_{j,}$$

where $\mu$ is the overall mean, ${\alpha }_{j,t}$ is the season modelled by the trigonometric model shown below, ${\delta }_{j}$ is the environmental herd-section effect of the building in which the pigs are housed, ${\beta }_{n}$ is the effect of group size ${n}_{j}$, ${\beta }_{age}$ is the effect of the mean age ${\tau }_{j}$ of pigs in the group, ${\beta }_{w}$ is the effect of the mean weight ${w}_{w,j}$ of pigs in the group, ${\beta }_{sd}$ is the effect of the standard deviation of weight ${w}_{sd,j}$ in the group, ${\beta }_{d}$ is the effect of the mean DBV given by ${a}_{d,j}$ of pigs in the group, ${\beta }_{s}$ is the effect of the mean SBV given by ${a}_{s,j}$ of pigs in the group, and ${\beta }_{ssd}$ is the effect of the interaction between ${w}_{sd,j}$ and ${a}_{s,j},$ given by ${\delta }_{s,sd,j}$, and ${e}_{j}$ is the residual ${e}_{j}\sim n(0,{\sigma }_{e}^{2})$. Note that Model (1), in addition to the effect of SBV, also includes an effect of DBV, which was necessary to account for the correlation between SBV and DBV. The mean levels of DBV and SBV denoted by ${a}_{d,j}$ and ${a}_{s,j}$ were obtained from predicted breeding values of the crossbred progeny that are equal to the averages of purebred parents’ predicted breeding values, as these were the only predicted breeding values available at the time when assigning piglets to high or low groups.

Seasonal effects were modelled by the following linear and trigonometric model:

$${\alpha }_{j,t}={b}_{0}t+{b}_{1}{\mathrm{sin}(\varphi }_{t})+{b}_{2}{\mathrm{cos}(\varphi }_{t}),$$

where $t$ is the experimental time recorded in weeks during the entire experimental period, ${b}_{0}$ is an adjustment for a linear trend during the experimental period, and the two coefficients ${b}_{1}$ and ${b}_{2}$ account for seasonal variation across the year since ${\varphi }_{t}=\frac{2\pi t}{52}$. The use of a trigonometric model reduces the number of parameters in Model (1) and ensures that only a linear trend and seasonal variation covering only a single period across a year are modelled with the same level of seasonal variation repeated in the second year. Note that if the traditional effects of month or season had been used instead of the above, more complex seasonal variation would have been introduced in the models, which would have created confounding between the seasonal effect and the time at which specific sires and dams were used. Such confounding would reduce the size of the genetic effects of DBV and SBV that are described by the estimated levels of ${a}_{d,j}$ and ${a}_{s,j}$.

Since all models on the group level were assumed to be linear and follow a Gaussian distribution, Model (1) was estimated by least squares using the lm function in R and the ANOVA test was used to test the significance of effects.

#### Individual pig level analyses

At the individual pig level, the behavioural traits were analysed using ordinal logistic regression. The traits considered were the number and the severity of the ear lesion and tail biting injuries, and behaviour in the weighing crate. Let $\mathrm{Y}$ be an ordinal outcome of the behavioural trait with $k$ categories. Then, $p(\mathrm{Y}\le k)$ is the cumulative probability of $\mathrm{Y}$ being lower than or equal to a specific category $k$ = 1, 2, …, $k$ −1. The odds of being lower than or equal to a category $k$ can be defined as:

$$\mathrm{log}\left(\frac{p(\mathrm{Y}\le k)}{p(\mathrm{Y}>k)}\right)=logit\left(p\left(\mathrm{Y}\le k\right)\right).$$

Then, letting ${y}_{ij}$ be the observed category of $\mathrm{Y}$ for pig $i$ in group level $j$, the ordinal logistic regression model was obtained by the expectation:

2 $$logit\left(p\left({y}_{ij}\le k\right)\right)={\alpha }_{j,t}+{\delta }_{j}+{\beta }_{n}{n}_{j}+{\beta }_{age}{\tau }_{j}+{\beta }_{w}{w}_{w,j}+{\beta }_{sd}{w}_{sd,j}+{\beta }_{d}{a}_{d,j}+{\beta }_{s}{a}_{s,j}+{\gamma }_{j},$$

where ${\alpha }_{j,t}$ includes linear and trigonometric year-season effects similar to the group levels $j$ in Model (1), ${\delta }_{j}$ is the environmental herd-section effect of the building in which the pigs are housed, ${\beta }_{n}$ is effect of group size, ${\beta }_{age}$ is effect of mean age, ${\beta }_{w}$ and the ${\beta }_{sd}$ are effects of mean body weight and of the standard deviation of body weight, ${\beta }_{s}$ is effect of mean group SBV level, ${\beta }_{d}$ is effect of the mean group DVB level, and ${\gamma }_{j}\sim N(0,{\sigma }_{\gamma }^{2})$ denotes the environmental random group effect in group $j$ to which pig $i$ belongs. Significance of the random group effect was tested using a likelihood ratio test by comparing models with and without the effect in Model (2).

Few pigs had three or more ear lesions. Therefore, for the analysis at the individual pig level, the five-category score of number of ear lesions was reduced to three categories and $k$ = 2, i.e., 0, 1, > 1. When analysing ear lesion severity, only pigs with ear lesions were analysed, i.e., $k$ = 0 was missing, and number of ear lesions only had two categories (1, and > 1) to estimate the relation between number and severity of ear lesions.

All ordered logistic Models (2) were estimated by a maximum likelihood procedure using the R-Package “Ordinal” [20].

## Results

After one day in the finisher unit, 1876 pigs had no ear lesions (score 1), and 2852 pigs had one or more ear lesions scored into categories 2 to 5 (Table 3). After seven weeks in the finisher unit, only 477 of 4470 pigs had ear lesions. Among the 2852 pigs that had ear lesions after one day in the finisher unit, the most frequently observed severity score was 2 (n = 1733 pigs; Table 4). Only 225 pigs of these 2852 had three ear lesions at day 1 and 38 pigs had four ear lesions. Therefore, the pigs were grouped into three levels of ear lesions, i.e. 0, 1, and ‘ > 1’, which resulted in a more balanced distribution of 1876, 1050, and 1802 pigs in each of the three response levels (Table 4).

**Table 3:** Frequencies of pigs across behavioural trait scores at day 1 and week 7after entering the finishing unit

Trait (below) \ Score (to the right)	1	2	3	4	5	Sum
Ear lesions* after 1 day	1876	1050	1539	225	38	4728
Ear lesions* after 7 weeks	3993	262	189	19	7	4470
Tail biting injuries** after 1 day	4002	590	47	89	–	4728
Tail biting injuries** after 7 weeks	3926	451	24	122	–	4523
Response to weighing crate*** after 1 day	421	2690	1607	9	–	4727
Response to weighing crate** after 7 weeks	473	2812	1184	1	–	4470

*The scores 1, 2, 3, 4, and 5 indicate the recorded number of 0, 1, 2, 3 and > 3 ear lesions

**The score = 1 indicates no tail biting injuries and score values > 1 indicate increasing severities of tail injury

***The scores 1, 2, 3, and 4 indicate “pig stands still during weighing and appears calm”, “pig moves around during weighing”, “pig moves around a lot during weighing, jumping and crashing into crate fittings”, and “pig appears ‘frozen with fear’ and stands still during weighing” and are on the ordinal scale. Scores 3 and 4 were combined in the analyses due to the scarcity of pigs that received score 4

**Table 4:** Number of pigs [percentages] with different combinations of ear lesion severity and number of ear lesions per pig at day 1 after entering the finisher unit

Severity	0 lesions	1 lesion	> 1 lesion	Sum
Score 1	–	257 [24.5]	377 [20.9]	634
Score 2	–	712 [67.8]	1021 [56.7]	1733
Score 3	–	81 [7.7]	404 [22.4]	485
Not scored	1876	–	–	1876
Sum	1876	1050 [100.0]	1802 [100.0]	4728

After one day in the finisher unit, 4002 pigs had no tail biting injuries and 726 had some degree of tail biting injury (Table 3). Among these 726 pigs, 590, 47, and 89 pigs had tail biting injuries that fell into the severity categories 2, 3, and 4, respectively. After seven weeks in the finisher unit, 451, 24, and 122 pigs had tail biting injuries that fell into the severity categories 2, 3, and 4, respectively. For tail biting injuries, we included 53 pigs which had been removed from the experiment by farm staff because of very severe tail biting injuries. These 53 pigs were assigned to the severity category 4. Thus, after seven weeks, there were 4470 + 53 = 4523 pigs that had a record of tail biting, but 4470 pigs that had records for the other behavioural traits (see Table 3).

For behaviour in the weighing crate one day after the pigs were moved to the finisher unit, 421, 2690, 1607, and 9 pigs received scores 1 (calm), 2, 3, and 4 (frozen with fear), respectively, out of 4727 pigs (Table 3). After seven weeks in the finisher unit, the behaviour in the weighing crate was recorded on 4470 pigs, of which 473, 2812, 1184 and 1, received scores 1, 2, and 3 or 4, respectively (Table 3). In total, only 10 pigs received the highest score of 4 (frozen with fear) (9 pigs after 1 day and 1 pig after 7 weeks) and, hence, scores 3 and 4 were combined for the analysis.

**Fig. 1:**
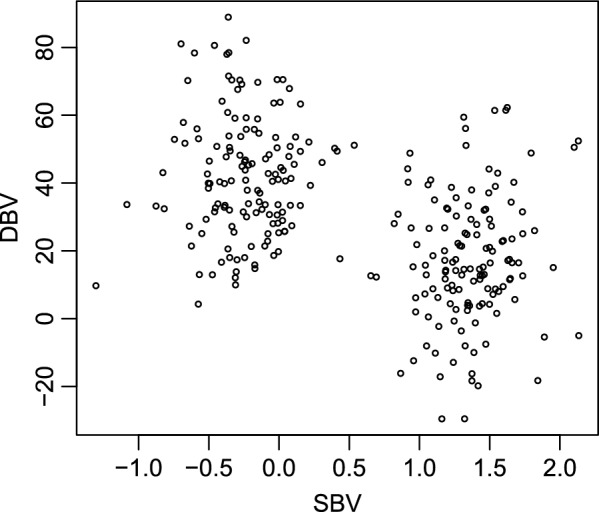
Relationship between direct (DBV) and social (SBV) breeding values of crossbred pigs per group (pen). The overall mean of low and high SBV across groups (experimental pens) were − 0.23 and 1.36 and the associated overall means of DBV were respectively 42.2 and 17.0

At the group level, Fig. 1 shows a clear distinction between the groups classified as having a high mean SBV (1.36) and those classified as having a low mean SBV (− 0.23). In addition, the groups of pigs with low SBV had a high mean DBV, i.e. 42.2, and conversely, the groups of pigs with high SBV had a low mean DBV, i.e. 17.0 (Fig. 1). This correlation between SBV and DBV indicates that all the following analyses and considered models must include the effects of both SBV and DBV.

### Statistical results at the group level

**Fig. 2:**
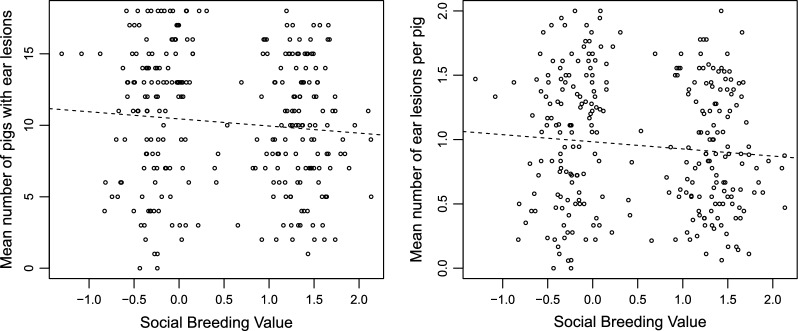
Relationship between ear lesions and mean social breeding value per group (pen). One day after the pigs entered the finisher unit, effect of the mean social breeding value (SBV) in the groups on (1) the mean number of pigs within the group afflicted with ear lesions, and (2) the mean number of ear lesions per pig within the group

After one day in the finisher unit, the groups with high SBV had fewer pigs with ear lesions than the groups with low SBV (p = 0.049). The estimated regression coefficient was − 0.50 (Table 5), which suggests a favourable relationship between SBV and the number of pigs exhibiting signs of ear lesions in the group. This means that, in groups for which the mean SBV of pigs increased by two units, there was one pig less with ear lesions. This favourable effect is also evident in the left panel of Fig. 2, where the two groups of dots show the two experimental groups (high or low SBV) and the regression line shows the effect of SBV, with an estimated slope of − 0.5.

**Table 5:** Means, estimates and significance levels for the effects of standard deviation (SD) of body weight, group mean of the social breeding value (SBV), and group mean of the direct breeding value (DBV) on different traits based on model (1)

Traits	Mean	SD of weight		SBV		DBV	
		Estimate	p-value	Estimate	p-value	Estimate	p-value
Mean number of pigs having ear lesions per group	10.5	− 0.09	0.76	− 0.50	0.049	0.010	0.50
Mean number of ear lesions per pig	0.98	− 0.008	0.86	− 0.06	0.035	0.0014	0.40
Mean number of pigs having tail biting injuries per group	2.2	0.187	0.04	0.002	0.83	− 0.002	0.75

The mean number of ear lesions per pig was significantly smaller (p = 0.04) in the groups with a high mean SBV and the estimated regression coefficient for SBV was − 0.06 (Table 5). Thus, with a 1 unit increase in the mean SBV of a group, the mean number of ear lesions per pig decreased by 0.06. The favourable effect of SBV on the group mean number of ear lesions per pig is illustrated in the right panel of Fig. 2. The results were obtained with an average group size of 17.4 pigs per pen. After seven weeks in the finishing unit, no significant effect of SBV on ear lesions was found.

Tail biting injuries recorded after one day in the finishing unit were not affected by SBV. Similarly, after seven weeks in the finishing unit, the association between group mean tail biting injuries and group mean SBV was not statistically significantly different from zero (Table 5). However, for tail biting injuries, an increase in the standard deviation of start body weight in the group increased the occurrence of tail biting injuries in the groups (p = 0.04), as shown by the estimated regression coefficient of 0.187 (Table 5). The direct effect of mean body weight in the group was not significant for tail biting or for the mean number of pigs with ear lesions or for the number of ear lesions per pig within the group.

### Statistical results at the individual pig level

After one day in the finisher unit, the mean SBV in the groups significantly affected the number of ear lesions per pig at the individual pig level (p = 0.014), with an estimated regression coefficient for SBV of − 0.094 (Table 6). This agrees with the direction of the effect of SBV at the group level with estimates of − 0.50 and − 0.06 (Table 5). Thus, the favourable effect of SBV was supported both at the group and the individual pig level data. Although the estimated regression coefficient of 0.004 was small, the individual number of ear lesions per pig was also significantly (p = 0.021) but unfavourably affected by the mean DBV of the group (Table 6). The results for ear lesions at the individual level (Table 6) show that the random group variation was not significant, then we redid the analysis without this effect, i.e., for ${\gamma }_{j}=0$, and it shows that no interaction between pigs within the group was found for this trait.

**Table 6:** Estimates and significance levels for the effects of individual body weight, age at about 30 kg, group mean of the social breeding value (SBV), group mean of the direct breeding value (DBV), and the estimated variance of the random group effect on different traits based on model (2) using individual pig level recordings

Traits	Body weight		Age		Number of ear lesions		SBV		DBV		Group variance
	Estimate	p-value	Estimate	p-value	Factor	p-value	Estimate	p-value	Estimate	p-value	${\sigma }_{\gamma }^{2}$
Number of ear lesions, day 1	− 0.038	< 0.001	0.0224	< 0.001	–	–	− 0.094	0.014	0.004	0.021	0
Severity of ear lesions conditional upon the number of ear lesions, day 1	− 0.022	0.006	0.0306	0.0004	0.518	< 0.0001	− 0.221	0.05	− 0.0052	0.25	1.24
Tail biting (present or not), day 1	0.0097	0.19	–	–	–	–	0.103	0.26	− 0.0009	0.80	0.59
Tail biting (present or not), week 7	− 0.002	0.77	–	–	–	–	0.011	0.91	0.0011	0.78	0.74
Behaviour in the weighing crate, day 1	− 0.029	< 0.0001	–	–	–	–	− 0.03	0.61	− 0.003	0.26	0.36
Behaviour in the weighing crate, week 7	− 0.006	0.32	–	–	–	–	− 0.146	0.027	− 0.007	0.008	0.34

The severity of the ear lesions at day 1 in the finisher unit (Table 6) depended significantly (p < 0.0001) on the number of ear lesions, with an increase in the severity of ear lesions by 0.518 when the number of ear lesions increased from 1 to > 1. This gives an odds ratio equal to exp(0.518) = 1.68, indicating that an increase in the number of ear lesions from 1 to > 1 increased the proportion of pigs with a higher severity score level. The most significant change in severity score was found for severity score 3. If the number of ear lesions increased from 1 to > 1, the number of animals with a severity score of 3 increased from 7.7 to 22.4% (Table 4).

The severity of ear lesions at day 1 conditional upon the number of ear lesions at day 1 was also affected by the mean SBV of the group (p = 0.05, Table 6), with an estimate of − 0.221 showing that 1 unit increase in the mean SBV of pigs in the pen resulted in a reduction in the severity score odds ratio, i.e., exp(− 0.221) = 0.80. It shows that the proportion of pigs that were observed with a lower severity score was increased by 20% on a three-level scale.

The relationship between SBV and ear lesions after seven weeks in the finisher unit was not statistically significant and is, therefore, not shown. The individual score level of tail biting was not affected by the mean SBV in groups neither after one day (p = 0.26) nor after seven weeks (p = 0.91) in the finisher unit (Table 6).

The response to weighing at day 1 was significantly affected by individual body weight of the pigs (p < 0.0001, Table 6). The negative estimate of − 0.029 shows that the larger pigs received a lower score, indicating greater calmness during weighing. The response to weighing at day 1 was not significantly (p = 0.61) affected by mean SBV in the group (Table 6).

The response to weighing at week 7 was significantly affected by the mean SBV in the group (p < 0.027, Table 6) with the negative estimate (− 0.146) indicating a favourable relationship since lower values indicate calmer pigs. The odds ratio exp(− 0.146) = 0.86 was less than 1, and thus, if the mean SBV in the group increased by 1 unit, then the proportion of pigs that obtained a calmer score level was increased by 14%. In addition, DBV favourably affected the behaviour in the weighing crate at week 7 (p = 0.008, Table 6), with the negative estimate (− 0.007) showing that an increase in the mean DBV in the group by 1 unit resulted in 100(1-exp(− 0.007)) = 0.7% decrease in the proportion of pigs receiving a lower score level. This indicates that groups of crossbred pigs with high group means of both SBV and DBV were calmer in the weighing crate compared to groups with low group means of SBV and DBV.

## Discussion

In this study, our aim was to investigate if selection on SBV for growth rate in purebred lines has a positive effect on behavioural traits in their crossbred progeny. The results show that, at day 1 after the crossbred pigs had entered the finisher unit, significant effects of selection on SBV were observed (Table 5). Compared to groups of pigs with low SBV, groups of pigs with high SBV had both fewer pigs with ear lesions and a smaller number of lesions per pig. An increase in the mean SBV of the group by 1 unit reduced the mean number of ear lesions per pig by 6% (Fig. 2) and the proportion of pigs with a lower level of severity was increased. After seven weeks in the finishing unit, an increase in the mean SBV of the group by 1 unit reduced the proportion of pigs having an increased score for response to behaviour in the weighing crate by 14%. Thus, our results show that selection on SBV for growth rate in purebred lines has a beneficial effect on ear lesions at day 1 after their crossbred progeny entered the finisher unit and on the behaviour in the weighing crate after seven weeks in the finisher unit with a calmer behaviour. However, for ear lesions after seven weeks in the finisher unit and for tail biting after one day and seven weeks in the finisher unit, the effect of selection on SBV for growth in the purebreds was not statistically significant. Thus, our starting hypotheses are only partly validated. However, it should be noted that tail biting behaviour varies greatly between farms and over time. In this study, the large majority of the pigs displayed no tail biting injuries. If we had performed our study using a larger population size or on a farm where tail biting was more frequent, it is possible that positive effects of selection on SBV in the purebreds would have been detected.

### Effect of SBV

The effect of group mean SBV on the mean number of pigs receiving ear lesions within the group are the simplest to interpret. These results showed a favourable effect of SBV on the number of pigs exhibiting ear lesions in the group and the mean number of ear lesions per pig (Table 5). Group level data were calculated from individual animal records of injuries, which were affected by weight and age. However, ear lesions were based on counts on an ordered scale, which resulted in a binary data structure. Therefore, analyses at the individual pig level assuming ordinal data may provide a more accurate estimation of the effect of SBV on incidence of injuries. The low but statistically significant effect of SBV at the group level (Table 5) was supported by an increased level of significance of SBV at the individual animal level (Table 6). In Model (2), the correlation between pigs receiving ear lesions within groups was found to be non-significant and was therefore ignored by setting the random group effect to zero (${\gamma }_{j}=0$). Furthermore, Model (2) was expanded to examine the interaction between the number and maximum severity of ear lesions. Based on this analysis, an increase in SBV by 1 unit significantly decreased the maximum severity of ear lesions, conditional upon the number of lesions, by 0.22 (Table 6). Therefore, we conclude that there was a favourable effect of SBV both on the number and the severity of ear lesions.

The favourable effect of SBV on ear lesions found in this study agrees with the findings in other studies about the relationship between SBV and ear biting [8, 9]. Camerlink et al. [8] found that crossbred pigs aged 8 to 23 weeks with high SBV for growth spent 27% less time on ear biting of pen mates than pigs with low SBV, and Hong et al. [9] showed that groups of crossbred pigs with high SBV had a significantly lower frequency of agonistic behaviours, including biting after mixing. These two studies had smaller group sizes, i.e. six pigs per pen and the pigs were housed under experimental conditions compared to those in our study, where pigs were housed under commercial conditions. Thus, all three studies support that selection on SBV in purebred pigs will result in reduced ear lesions in groups of crossbred pigs. Our results also support the favourable effect of SBV of F1-crossbred hybrids of DanBred Landrace and DanBred Yorkshire shown by Ask et al. [17].

We did not find a significant effect of mean SBV on the number of pigs with tail biting injuries in groups (Table 5) or on the individual score level of tail biting, recorded as present or absent (Table 6). This lack of statistical significance is likely due to the fact that we had too few animals with tail biting injuries. After one day in the finisher unit, the number of pigs with tail biting injuries (726) was much smaller than the number of pigs with ear lesions (2852). In Camerlink et al. [8], a significantly lower tail damage score was found in high SBV groups compared to low SBV groups in the finishing period. The frequency and severity of tail biting vary greatly between farms and over time within the same farm, making it a challenging behavioural trait to study [21, 22]. In our study, the limited number of pigs with tail injuries compared to the number of pigs with ear lesions affected the statistical power in the experiment and thus the possibility to identify a significant relationship between SBV and tail biting. We only found a slightly significant effect on tail biting injuries of the standard deviation in body weight at day 1 in the finisher unit (p = 0.039), with an estimated regression coefficient of 0.187 (Table 5). This indicates that tail biting is more frequent in groups with large variation in body weight, which implies that these groups contain small pigs. In some studies, (e.g. Beattie et al. [1]) small pigs have been shown to display a disproportionately large amount of tail biting behaviour, although body weight has not been found to be a risk factor for giving or receiving tail biting in other studies [1, 23]. Although we were not able to confirm an effect of SBV on tail biting injuries, it is noteworthy that Brunberg et al. [24] showed that the same individuals tend to show both ear and tail biting. If tail biting had been more common and severe in our experimental data, a favourable effect of SBV on tail biting injuries may have been found, in line with the study of Camerlink et al. [8].

Response to weighing after 7 weeks in the finisher unit was significantly affected by SBV. Our results agree with other studies that show that pigs with high SBV may be less fearful and better at handling stressful situations [25–27]. The groups of crossbred pigs with high SBV showed a significantly calmer reaction to the weighing crate compared to groups with low SBV. However, the effect was not evident after one day in the finisher unit, which may be due to instability of the social group at that time, stress of movement to the new accommodation, or due to their younger age. It is known that major disruptions to management and physiology can cause changes in the response of animals to potentially threatening stimuli [28]. Pigs become more challenging to handle as they grow older and a reduction in aversive responses to restraint in older pigs due to increased SBV likely reflects an improvement in animal welfare and handling ease. In conclusion, our results indicate that selection on SBV for growth rate is expected to increase calmness of pigs in weighing crates after 7 weeks in the finishing unit.

Social genetic effects often operate through social behaviour, as this is a key mechanism by which one individual can affect the phenotype of another [10, 29, 30]. In spite of this, the social behaviours responsible for social genetic effects have been poorly studied. Our analysis shows that a higher SBV is associated with less harmful behaviour, as indicated by ear lesions. Other social behaviours may also contribute to SBV. Competition for limited resources and aggression are expected to be major determinants of SBV [10, 29, 30], although the effect of prosocial behaviours on transmission of social genetic effects has not been studied. Using the same population of animals as Camerlink et al. [8], it has been shown that pigs selected for high SBV exhibit less one-sided attacks (i.e. non-reciprocal biting) and less aggression upon reunion with familiar pen mates after a 24-h period of separation [25]. Canario et al. [10] also reported an effect on aggressive behaviour i.e. that pigs selected for higher total breeding value (TBV) for growth, which includes both DBV and SBV, did not differ from other pigs in their DBV for lesions on the anterior part of the body but had a lower DBV for posterior lesions. The motivations that underlie tail and ear biting, on the one hand, and aggressive fighting, on the other hand, are believed to differ. Tail and ear biting are understood to reflect redirected foraging behaviour in an unstimulating physical environment in the majority of cases [31], while aggressive fighting occurs to establish and thereafter maintain dominance relationships [32]. Therefore, it is difficult to extrapolate how selection on SBV may affect aggressive fighting based on its effects on tail and ear biting. Nevertheless, in the light of the existing literature, our work suggests that SBV are likely the result of a range of social behavioural traits.

### Effect of DBV

In the present study, we accounted for the correlation between SBV and DBV (see Fig. 1) by designing the experiment as a balanced block design. However, in 15 of the 72 weeks, only three experimental groups per block were available and for these weeks it was decided not to create groups of high and low DBV. Hence, in those cases, we prioritised the effect of SBV over the effect of DBV. However, the mean effect of DBV of the groups was accounted for in the analyses.

The mean DBV of the group had a significant (p = 0.021) but unfavourable effect on the individual number of ear lesions per pig (Table 6). However, this was not supported by the results from the linear normal models at the group level, neither by the effect of DBV on the mean number of pigs with ear lesions in the group (p = 0.50) nor by the mean number of ear lesions per pig within groups (p = 0.40) (Table 5).

We also found that DBV favourably affected behaviour in the weighing crate at week 7 (Table 6). When mean DBV in the group increased by 1 unit, the proportion of pigs with a lower score level was increased by 0.7%. Although the corresponding one-unit effect of SBV was much larger at 14%, the 0.7% for DBV may still be important in practical selection since the standard deviation of DBV was about 30 times larger than the standard deviation of SBV, as shown in Fig. 1.

### Implication of social genetic effects in pig breeding

The unfavourable correlation between DBV and SBV (see Fig. 1) implies that traditional breeding goals in pig breeding, i.e. selection for improved growth, but with no selection pressure for improving social behaviour, is slowly and indirectly selecting for pigs with lower SBV, which most likely operates through expression of less favourable social behavioural traits. Thus, ethologists and quantitative geneticists have for decades been working towards identifying efficient methods to breed for improved social behaviour [6, 8, 10]. In order to improve pig welfare through improved social behaviour, selective breeding using a combination of DBV and SBV is one alternative selection strategy compared to selecting animals for traits related to social behaviour only on their breeding values predicted by a classical animal model. Selecting animals only on breeding values predicted by such a classical animal model of social behavioural traits is ineffective, because although behavioural traits are strongly heritable, they are also complex and difficult and very time-consuming to phenotype on a large-scale [33]. In practical pig breeding, the focus has been on quantifying and reducing damaging behaviours. With selection on direct breeding values for traits related to social behaviour, this implies selection of pigs with, e.g., the fewest ear lesions or tail bites. However, it would be more useful to select the pigs that do not perform damaging behaviour. This study shows that SBV estimated for growth reflect damaging social interactions between pigs, and that selection for such SBV is expected to reduce the number of pigs with ear lesions, as well as the number of ear lesions per pig and their severity. In addition, our results show that selecting for SBV is expected to increase the calmness of pigs in weighing crates after seven weeks and, thereby, result in pigs which are calmer when handled by humans.

In the present study, the evaluation of the effects of selection on SBV were based on manual recordings by a technician, i.e., observations of ear lesions, tail biting injuries, and behaviour in weighing cates, and these recordings were assumed to reflect the animal behaviour in the pens. In the future, automated recordings based on camera technology and image analysis may allow for recording of behaviour traits. A review by Wurtz et al. [33] showed that most studies used standard digital colour video cameras for data collection, especially in group-housed pigs. The most common behaviours recorded from the reviewed studies were activity level, area occupancy, aggression, gait scores, and resource use. Although sample sizes were generally small for these studies, it indicates that this technology is rapidly developing and the future for collecting phenotypes on social behaviours is encouraging. Breeding for less damaging behaviour is expected to substantially improve animal welfare in group-housed pigs and contribute to more effective pig production. However, when selecting for growth rate, selection on predicted SBV for growth rate will continue to be relevant since it avoids the unfavourable behavioural outcomes that follow from selecting pigs only on their predicted DBV for growth rate. Furthermore, if selection on SBV becomes part of the breeding goal, additional animal behavioural traits may not need to be considered in the breeding goals, as the associated trend of unfavourable behavioural outcomes introduced by the unfavourable correlation to DBV is avoided.

## Conclusions

Our results show that mating purebred DanBred Landrace sows and DanBred Yorkshire boars with high SBV has a beneficial effect on ear manipulation at day 1 after their crossbred progeny enter the finishing unit, and that selection for SBV in the purebred lines reduces the number of ear lesions per crossbred pig housed under commercial conditions. In addition, after seven weeks in the finisher unit, calmer behaviour was observed in the weighing crate for pigs with high SBV. The incidence of tail bites was too low in this study to show how selection on SBV affects the number of pigs with tail biting injuries, either at the group level, or at the individual score level of tail biting. Overall, this study shows that selection of purebred lines on social breeding values for growth rate is expected to increase welfare in their crossbred progeny by reducing the number of ear lesions and by making these pigs easier to handle.

## Data Availability

The data analyzed during this study are not publicly available since they are owned by the Danish Agriculture & Food Council.
